# Supplementary material for: Transcriptional and post-transcriptional mechanisms modulate cyclopropane fatty acid synthase through small RNAs in Escherichia coli
Source: J Bacteriol. 2024 Jul 9;206(8):e00049-24. doi: 10.1128/jb.00049-24 (PMC11340327; doi:10.1128/jb.00049-24)
Supplement: Supplemental material — Figures S1 to S3; Tables S1 and S2. [file jb.00049-24-s0001.pdf]

Supplementary material

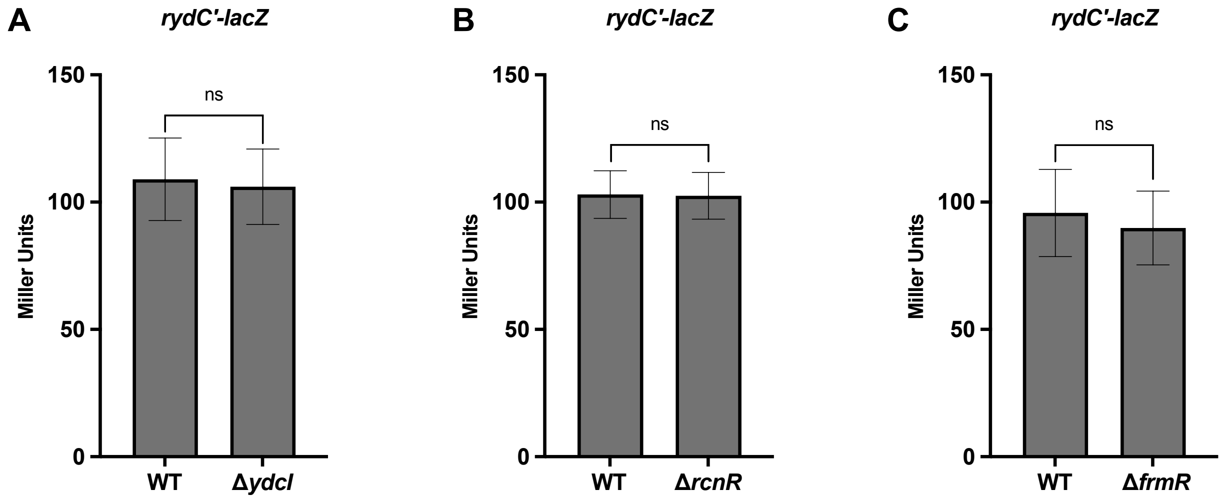

Figure S1. FrmR, RcnR and YdcI do not regulate *rydC* transcription. Strains harboring a *rydC'-lacZ* transcriptional fusion with deletions of indicated candidate regulators were constructed as described in Materials and Methods. Strains were grown overnight in TB then subcultured 1:100 to fresh TB medium. Subcultures were grown at 37°C with shaking for 3 hours and then β-galactosidase assays were performed. Error bars are standard deviations of the results of three independent experiments. Statistical significance of fusion activity in WT background compared to mutant backgrounds was determined using a two-tailed unpaired t-test, (ns, P not significant.)

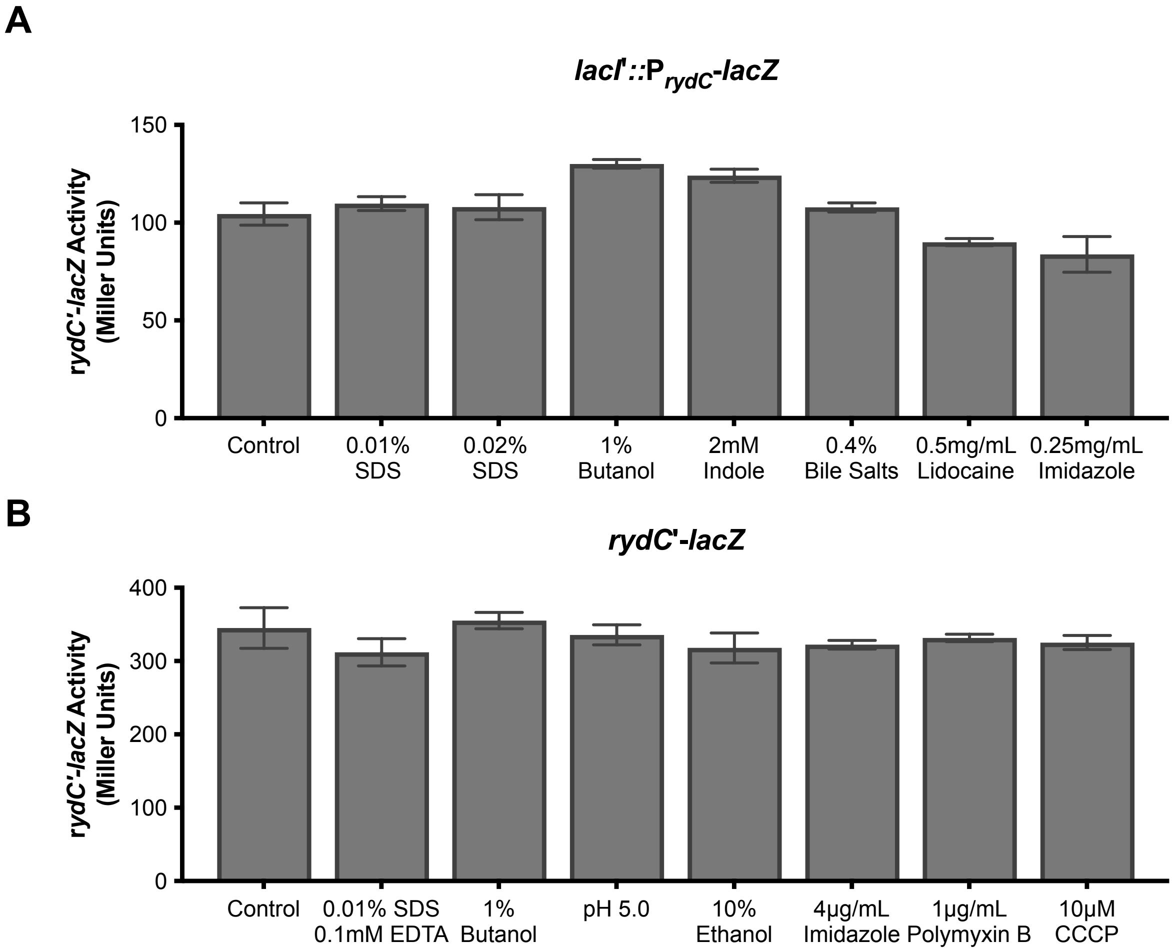

Figure S2. Membrane-disrupting stresses do not activate *rydC* transcription. Two *rydC* transcriptional fusions were constructed as described in the materials and methods section. Strains carrying a *rydC* transcriptional fusion were grown in TB medium to early exponential phase then cells were subcultured into TB medium containing various compounds at the indicated concentrations. Samples were harvested 20 minutes later and assayed for β-galactosidase activity of the reporter fusion. The error bars represent standard deviations of three independent experiments.

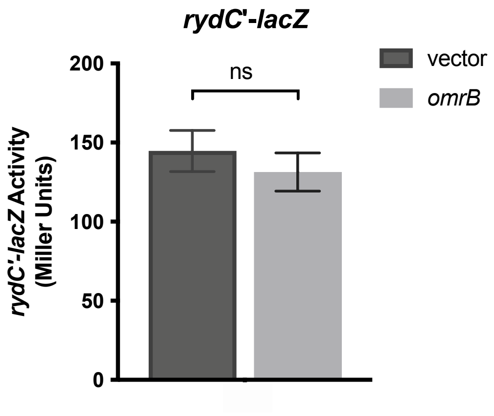

Figure S3. Expression of *omrB* does not alter *rydC* transcription.
A chromosomal *lacZ* transcriptional fusion was constructed at the native RydC locus (*rydC'-lacZ*) and its activity was assayed in response to expression of empty vector or *omrB*. Statistical significance of fusion activity in response to empty vector expression compared to *omrB* expression was determined using a two-tailed unpaired t-test, (ns, P not significant).

Table S1. Plasmids and strains used in this study.

**Plasmid**	**Vector**	**Genotype**	**Source or Reference**
pBRCS12	pHDB3	vector control	Wadler *et al.*, 2009
pCB15	pBRCS12	P_{lac}-*yieP (E. coli)*	This study
pCB7	pBRCS12	P_{lac}-*rydC*	This study
	pBRCS12	P_{lac}-*cfa*	This study
pCB16	pBRCS12	P_{lac}-*fnrS*	This study
pCB17	pBRCS12	P_{lac}-*fnrS-M*	This study
pCB18	pBRCS12	P_{lac}-*cyaR*	This study
pCB19	pBRCS12	P_{lac}-*ryhB*	This study
pCB20	pBRCS12	P_{lac}-*yieP (Salmonella)*	This study
pCB5	pBRCS12	P_{lac}-*cpxQ*	This study
pCB21	pBRCS12	P_{lac}-*omrB*	This study
pCB22	pBRCS12	P_{lac}-*omrB-M*	This study

**Strain**	**Parent**	**Genotype**	**Source or Reference**
DJ480	MG1655	Δ*lac* X74	D. Jin, NCI*
PM1205	PM1203	*lacI'*:: P_{BAD}-*cat-sacB-lacZ*, miniλtet^{R}, Δ*araBAD araC*+, *mal::lacI*^{q}	Mandin *et al.,* 2009
DJ624	DJ480	Δ*lac* X74 *mal::lacI*^{q}	D. Jin, NCI
CB121	PM1205	*lacI'*:: P_{rydC}-*lacZ*, miniλtet^{R}, Δ*araBAD araC*+, *mal::lacI*^{q}	This study
CB308	DJ624	P'*yieP-lacZ*	This study
CB438	DJ480	Δ*lac* X74 λ*att*B::*lacI*^{q}	This study
CB536	CB438	Δ*lac* X74 *mal::lacI*^{q} Δ*rydC*	This study
CB565	CB4238	Δ*lac* X74 *mal::lacI*^{q} Δ*cfa*	This study
CB227	DJ624	Δ*lac* X74 *mal::lacI*^{q} *rydC'-lacZ*	This study
CB273	DJ624	Δ*lac* X74 *mal::lacIq* Δ*yieP rydC'- lacZ* in locus	This study
CB429	14028S	*rydC'- lacZ* pKD136 in Sa 14028S	This study
CB432	14028S	Δ*yieP rydC'- lacZ* pKD136 in Sa 14028S	This study
CB268	DJ624	Δ*lac* X74 *mal::lacI*^{q} Δ*yieP*	This study
AK27	PM1205	Δ*araBAD araC*+, *mal::lacI*^{q} P_{BAD}-*cfa'-'lacZ*-Long	King *et al.,* 2019
CB259	AK27	Δ*araBAD araC*+, P_{BAD}-*cfa'-'lacZ*-Long Δ*yieP*	This study
CB631	CB259	Δ*araBAD araC*+, P_{BAD}-*cfa'-'lacZ*-Long Δ*yieP rydC::kan*	This study
CB588	AK27	Δ*araBAD araC*+, P_{BAD}-*cfa'-'lacZ*-Long Δ*rydC*	Bianco et al. 2019
CB308	DJ624	Δ*lac* X74 *mal::lacI*^{q} *yieP'-lacZ*	This study
CB1106	PM1205	Δ*araBAD araC*+, P_{BAD}-*yieP'-'lacZ*	This study
CB1114	PM1205	Δ*araBAD araC*+, P_{BAD}-*yieP'-'lacZ-M*	This study
NC5	DJ480	Δ*lac* X74 Δ*cpxR*	This study
NC6	NC5	Δ*lac* X74 Δ*cpxR rydC'-lacZ*	This study
NC11	DJ480	Δ*lac* X74 Δ*rcnR*	This study
NC13	NC11	Δ*lac* X74 Δ*rcnR rydC'-lacZ*	This study
NC15	DJ480	Δ*lac* X74 Δ*ydcI*	This study
NC16	NC15	Δ*lac* X74 Δ*ydcI rydC'-lacZ*	This study
CB331	PM1205	*lacI'*:: PyieP-*lacZ*, miniλtet^{R}, Δ*araBAD araC*+, *mal::lacI*^{q}	This study
CB139	DJ480	Δ*lac* X74 Δ*baeR rydC'-lacZ*	This study
CB176	DJ480	Δ*lac* X74 Δfur *rydC'-lacZ*	This study
CB179	DJ480	Δ*lac* X74 Δ*rcsB rydC'-lacZ*	This study
CB180	DJ480	Δ*lac* X74 Δ*rcsF rydC'-lacZ*	This study
CB140	DJ480	Δ*lac* X74 Δ*baeS rydC'-lacZ*	This study

Table S1. Plasmids and strains used in this study, continued

CB183	DJ480	Δ*lac* X74 Δ*fadR rydC'-lacZ*	This study
CB222	DJ480	Δ*lac* X74 Δ*arcB rydC'-lacZ*	This study
CB223	DJ480	Δ*lac* X74 Δ*ompR rydC'-lacZ*	This study
CB224	DJ480	Δ*lac* X74 Δ*nadR rydC'-lacZ*	This study
CB274	DJ480	Δ*lac* X74 Δ*rpoS rydC'-lacZ*	This study
CB366	DJ480	Δ*lac* X74 Δ*pspA rydC'-lacZ*	This study
CB367	DJ480	Δ*lac* X74 Δ*pspB rydC'-lacZ*	This study
CB368	DJ480	Δ*lac* X74 Δ*pspC rydC'-lacZ*	This study
CB369	DJ480	Δ*lac* X74 Δ*pspD rydC'-lacZ*	This study
CB370	DJ480	Δ*lac* X74 Δ*pspE rydC'-lacZ*	This study
CB371	DJ480	Δ*lac* X74 Δ*pspF rydC'-lacZ*	This study
CB372	DJ480	Δ*lac* X74 Δ*pspG rydC'-lacZ*	This study
CB142	DJ480	Δ*lac* X74 Δ*rseA rydC'-lacZ*	This study
CB403	DJ480	Δ*lac* X74 Δ*pdhR rydC'-lacZ*	This study
CB175	DJ480	Δ*lac* X74 Δ*lrp rydC'-lacZ*	This study
CB400	DJ480	Δ*lac* X74 Δ*cra (fruR) rydC'-lacZ*	This study
NC19	DJ480	Δ*lac* X74 λ*att*B::*lacI*^{q} *frmR*:kan	This study
NC21	NC19	Δ*lac* X74 λ*att*B::*lacI*^{q} Δ*frmR*	This study
NC22	NC21	Δ*lac* X74 λ*att*B::*lacI*^{q} Δ*frmR rydC'-lacZ*	This study

Table S2. Oligonucleotide primers used in the study.

Oligo	Description	Sequence 5'-3'
GSP	Gene specific primer for 5'RACE of *yieP*	TTGTGGCATG ACCCGAGTAC CAATTCGCGG
GSP2	Gene specific primer for 5'RACE of *yieP*	TCGCGGTAAA ACCATCCCTT
GSP3	Gene specific primer for 5'RACE of *yieP*	TTGCCGTTAA CGTTTTGACC
TSO	Template switching oligo for 5'RACE of *yieP*	ACACTCTTTCCCTACACGACGCTCTTCCGATCTrGrGrG
OUTER	PCR primer for 5'RACE of yieP	AATGATACGGCGACCACCGAGATCTACACTCTTTCCCTACAC GACGCTCTTCCGATCT
INNER	PCR primer for 5'RACE of yieP	CTACACGACGCTCTTCCGATCT
RydC NB	Probe for RydC Northern blot	/5bioG/CGCCTGTACTAAAACCGACCCGTGGTACAGGCGAAGA ATACGGG
5S	Probe for 5S Northern blot	GTTTCACTTCTGAGTTCGGCATGGGGTCAGGTGGG
CB125	Forward primer for cloning E. coli rydC into pBRCS12 contains Bam site	CCCCCCCCGGATCCCTTCCGATGTAGACCCGTATTCTT
CB100	Reverse primer for cloning E. coli rydC into pBRCS12 contains HindIII site	CCCCCCCCAAGCTTCCGCGTAAACGTTCCTGAAGGATAT
CB316	Forward primer for cloning E. coli omrB into pBRCS12 contains Bam site	CCCCCCCCGGATCCcccAGAGGTATTGATAGGTG
CB317	Reverse primer for cloning E. coli omrB into pBRCS12 contains HindIII site	CCCCCCCCAAGCTT TGACCGGCaaaAAAAACCTG
CB4F	Forward primer to make in locus rydC lacZ fusion	ATGGTTTTATTTATCATACAAATAAATATAATAGGCGcttGTGTA GGCTGGAGCTGCTTC
CB4R	Reverse primer to make in locus rydC lacZ fusion	CGCATGATGCCGCGTAAACGTTCCTGAAGGATATTTAAAGAT GGGAATTAGCCATGGTCC
CB7	Forward primer to make lacI':: PrydC-lacZ	CTATGCCATAGCATTTTTATCCATAAGATTAGCGGATCCATAA CCTCCATGGTTTATCG
CB8	Reverse primer to make lacI':: PrydC-lacZ	TAACGCCAGGGTTTTCCCAGTCACGACGTTGTAAAACGACCA TAGCTGTTTCCTGTGTGACGGAAGCGCCTATTATATTT
CB127	Forward primer for cloning CpxQ into pBRCS12 contains Bam site	CCCCCCCCGGATCCTTTTCCTTGCCATAGACACCATCCC
CB106	Reverse primer for cloning CpxQ into pBRCS12 contains HindIII site	CCCCCCCCAAGCTTTGACGCTAGTATAACGGAAGC
CB47	Forward primer to make in locus yieP lacZ transcriptional fusion	CTTCCTGGTG GCATACTAAG GAGGAATAAA AAGAAAAAGG AAGATAAGTA GTGTAGGCTGGAGCTGCTTC
CB48	Reverse primer to make in locus yieP lacZ transcriptional fusion	CCATACTGCGCTTCTTTTTATCGCTCATTCAATGTCCTGTTATC GGGTTA ATGGGAATTAGCCATGGTCC
CB314	FnrS-M quick change forward	attgtgctatgcagtaattcaataaaggaagtaagacaatatggag
CB315	FnrS-M quick change reverse	ctccatattgtcttacttcctttattgaattactgcatagcacaat
CB254	Forward primer for cloning E. coli fnrS into pBRCS12 contains Bam site	CCCCCCCCGGATCC gcaggtgaatgcaacgtcaa
CB255	Reverse primer for cloning E. coli fnrS into pBRCS12 contains HindIII site	CCCCCCCCAAGCTT cgctgataaataacaggcaaaa
CBY1	Forward primer for cloning yieP 5'UTR into PM1205 to make PBAD -yieP'-'lacZ	ACCTGACGCTTTTTATCGCAACTCTCTACTGTTTCTCCAT GAGGAATAAAAAGAAAAAGG
CBY2	Reverse primer for cloning yieP 5'UTR into PM1205 to make PBAD -yieP'-'lacZ	TAACGCCAGGGTTTTCCCAGTCACGACGTTGTAAAACGAC CTTATCAGGTGAGCGTAGCA
CBY3	Forward primer for cloning yieP-M 5'UTR into PM1205 to make PBAD -yieP'-'lacZ	ACCTGACGCTTTTTATCGCAACTCTCTACTGTTTCTCCATGAG GAATAAAAAGATAAAGG

Table S2. Oligonucleotide primers used in the study.

CB129	Forward primer for cloning E. coli yieP into pBRCS12 contains Bam site	CCCCCCCCGGATCCGCATACTAAGGAGGAATAAAAAGAAAAAGG
CB130	Reverse primer for cloning E. coli yieP into pBRCS12 contains HindIII site	CCCCCCCCAAGCTTTGATAACCCGATAACAGGACATTGAATGAG
CB324	OmrB-M quick change forward	acccgaagttgacttcagctatcaatacctctggg
CB325	OmrB-M quick change reverse	Cccagaggtattgatagctgaagtcaacttcgggt
CB154	Forward primer for cloning Salmonella yieP into pBRCS12 contains Bam site	CCCCCCCCGGATCCatgCCTTTAAGCGCACAACAATTAG
CB155	Reverse primer for cloning Salmonella yieP into pBRCS12 contains HindIII site	CCCCCCCCAAGCTTTTAATTATCTGGTCTCTCATTGGGCG
CB66	Forward primer to make lacI':: PyieP-lacZ	CTATGCCATAGCATTTTTATCCATAAGATTAGCGGATCC TTGGTATTCA TTTTTCGTCT
CB67	Reverse primer to make lacI':: PyieP-lacZ	TAACGCCAGGGTTTTCCCAGTCACGACGTTGTAAAACGACCATA GCTGTTTCCTGTGTGA TACTTATCTTCCTTTTTCTT
CB78	Forward primer to make in locus rydC- lacZ transcriptional fusion in Salmonella	ATAGTAATTGCTTATTTTATTTGTCATACAAATAAGTATAATACCCGCTT GTGTAGGCTGGAGCTGCTTC
CB86	Reverse primer to make in locus rydC- lacZ transcriptional fusion in Salmonella	CTTAAGGCAAACAAGGCATCTATCAGAGGGGATGGCGTATTCCATACAGA CATATGAATATCCTCCTTAG
CB72	Forward primer to delete yieP in Salmonella using pKD13	CCAGGTGGCATACTAATAACCAAACGAAAGAGAAAGGAAGAACATTC ATGATTCCGGGGATCCGTCGACC
CB73	Forward primer to delete yieP in Salmonella using pKD13	CGCTTTTTTGCTGGTCATTCAATATCCTGTTAATTATCTGGTCTCTCATT TGTAGGCTGGAGCTGCTTCG
CB256	Forward primer for cloning E. coli *cyaR* into pBRCS12 contains Bam site	CCCCCCCCGGATCC gctGAAAAAC ATAACCCATA A
CB257	Reverse primer for cloning E. coli *cyaR* into pBRCS12 contains HindIII site	CCCCCCCCAAGCTTTATTTCATTGTATTACGCGT
CB226	Forward primer for cloning E. coli *ryhB* into pBRCS12 contains Bam site	CCCCCCCCGGATCC gcgATCAGGAAGACCCTCG
CB227	Reverse primer for cloning E. coli *ryhB* into pBRCS12 contains HindIII site	CCCCCCCCAAGCTT CCCGTGGATAAATTGAGAACGAAAGATC
CB1F	Forward primer for cloning E. coli *cfa* into pBRCS12 contains Bam site	CCCCCCCCGGATCC atgAGTTCATCGTGTATAGAAGAAGT
CB1R	Reverse primer for cloning E. coli *cfa* into pBRCS12 contains HindIII site	CCCCCCCCAAGCTT TTAGCGAGCCACTCGAAGGCC
NC_frmR_F	*frmR* deletion check primer forward	CATGCAGATGATGAGGTGCG
NC_frmR_R	*frmR* deletion check primer reverse	CTCTCGCTCTTCCTCAATATGG
